# Medical Society of the County of Erie

**Published:** 1900-06

**Authors:** Franklin C. Gram


### MEDICAL SOCIETY OF THE COUNTY OF ERIE.

MEMORIAL MEETING.

Reported by FRANKLIN C. GRAM, M. D., Secretary.

THE members of the Medical Society of the County of Erie, assembled in special session in the parlors of the Young Men's Christian Association of Buffalo, May 15, 1900, at 5 p.m., when the president, Dr. E. H. Ballou, announced the death of Dr. Louis P. Dayton, which occurred May 14, 1900, at the age of 80 years. He requested the society to take such action as it deemed appropriate.

On motion of Dr. Dambach, the chair appointed Drs. Howe, Slacer and Lothrop a committee to present a memorial. They submitted the following, which was adopted :

IN MEMORIAM.

The members of the Erie County Medical Society have heard with deep regret of the death of Dr. L. P. Dayton, and desire to place on record some tribute to his worth as a physician and a citizen. They recognise in him one who for many years occupied a prominent position in the profession and in the community. Not only was he skilled in his art, but his qualities of heart endeared him to his patients to an unusual degree. Moreover, he has left an excellent example of the physician's activity in municipal affairs and the value of such service. During the time that he occupied the position as mayor of the city he was earnest in fulfilling his duties conscientiously and thoroughly, and his interest in public hygiene at that time did much toward the establishment of the health department, which now reflects great credit on our city.

It is in recognition of these qualifications as a physician and as a man that we desire to pay this tribute—brief and imperfect though it be—to his name.

LUCIEN HOWE,

WM. H. SLACER,

THOMAS LOTHROP.

In speaking of the deceased, Dr. Wm. C. Phelps said that he had known Dr. Dayton for upwards of thirty years. Few members of the profession were better acquainted in the city or knew more people. The number of public offices he was called to fill was something phenomenal for a physician. In addition he took care of a large practice.

Drs. D. W. Harrington, Wm. H. Slacer, G. W. Pattison and J. J. Walsh also added brief, but eloquent tributes.

A telegram of condolence from Dr. Frederick F. Hoyer, of Tonawanda, was read by the secretary.

Adjournment followed.

